# Haemorrhage and Survival Times: Medical–Legal Evaluation of the Time of Death and Relative Evidence

**DOI:** 10.3390/diagnostics13040732

**Published:** 2023-02-15

**Authors:** Maricla Marrone, Loredana Bellantuono, Alessandra Stellacci, Federica Misceo, Maria Silvestre, Fiorenza Zotti, Alessandro Dell’Erba, Roberto Bellotti

**Affiliations:** 1 Dipartimento Interdisciplinare di Medicina, Legal Medicine Section, Università degli Studi di Bari Aldo Moro, I-70124 Bari, Italy; 2 Dipartimento di Biomedicina Traslazionale e Neuroscienze (DiBraiN), Università degli Studi di Bari Aldo Moro, I-70124 Bari, Italy; 3 Istituto Nazionale di Fisica Nucleare, Sezione di Bari, I-70125 Bari, Italy; 4 Dipartimento Interateneo di Fisica, Università degli Studi di Bari Aldo Moro, I-70126 Bari, Italy

**Keywords:** arterial lesions, time of haemorrhage, crime scene investigation

## Abstract

Haemorrhage is the name used to describe the loss of blood from damaged blood vessels (arteries, veins, capillaries). Identifying the time of haemorrhage remains a clinical challenge, knowing that blood perfusion of systemic tissues is poorly correlated with the perfusion of specific tissues. In forensic science, one of the most discussed elements is the time of death. This study aims to provide the forensic scientist with a valid model to establish a precise time-of-death interval in cases of exsanguination following trauma with vascular injury, which can be useful as a technical aid in the investigation of criminal cases. To calculate the calibre and resistance of the vessels, we used an extensive literature review of distributed one-dimensional models of the systemic arterial tree as a reference. We then arrived at a formula that allows us to estimate, based on a subject’s total blood volume and the calibre of the injured vessel, a time interval within which a subject’s death from haemorrhage from vascular injury falls. We applied the formula to four cases in which death had been caused by the injury of a single arterial vessel and obtained comforting results. The study model we have offered is only a good prospect for future work. In fact, we intend to improve the study by expanding the case and statistical analysis with particular regard to the interference factors to confirm its actual usability in practical cases; in this way, useful corrective factors can be identified.

## 1. Introduction

As is well known, the human body contains approximately 5–6 litres of blood, equal to about 1/12 of the body weight (considering an adult man weighing 70 kg and being 180 cm tall); this blood consists of a corpuscular part (45%) and a fluid part (55%). In the event of a rupture in a vessel of any type and calibre (arteries, veins, capillaries), a loss of blood called haemorrhage occurs. Depending on the component involved, there can be arterial, venous or capillary haemorrhage. It can be external, if the blood pours out of the body through natural breaches or orifices; it can be internal if, although leaving the injured vessel, it remains in the body.

Bleeding is divided into four classes by the American College of Surgeons’ advanced trauma life support (ATLS):

- Class I Haemorrhage: involves up to 15% of the blood volume.
- Class II Haemorrhage: involves 15–30% of total blood volume. The patient is often tachycardic. The body responds to blood loss with vasoconstriction, and skin is pale and cold to the touch.
- Class III Haemorrhage: involves the loss of 30–40% of the circulating blood volume. Blood pressure begins to decrease rapidly, heartbeats increase and a state of hypoperfusion of peripheral tissue (shock) occurs. Blood transfusion becomes necessary.
- Class IV Haemorrhage: results in loss of more than 40% of circulating blood volume. At this stage, the patient is in a coma.

Extensive haemorrhage, with a loss of 30–40% or more of the total circulating blood volume, leads to a state of hypovolaemic shock. Hypovolaemic shock is a condition of inadequate organ perfusion due to a loss of intravascular volume, resulting in a lowering of cardiac preload to a critical level and a reduction in macro- and microcirculation, with negative consequences on tissue metabolism and the triggering of an inflammatory reaction. The effects of shock are initially reversible but quickly become irreversible, causing multi-organ failure (MOF) and death [1]. From an etiopathogenetic point of view, blood can leak from vessels in three different ways: 1. rhexis; 2. diapedesis; 3. diabrosis. In each case, a haemorrhage can be the consequence of a clinical/pathological event or trauma. Traumatic injuries vary in severity. The most common types of traumatic injuries, the leading cause of avoidable death, include: abrasions (scratches); haematomas; lacerations; puncture wounds from objects, such as needles, nails or knives; crush and/or gunshot wounds [2].

The question the judge often asks the medical examiner in the case of an autopsy is the time elapsed between the traumatic event and death [3,4]. In the case of a haemorrhagic event, the answer to this question is not always easy to formulate [5]. In the case of a crime (whatever it may be) resulting in a copious haemorrhage, it is often difficult to answer the questions posed by the judge to the medical examiner: whether death was immediate or not, how long the victim survived and the possibility of acts performed by the victim after the injury, such as autonomy of movement or other conscious acts (e.g., asking for help, recognising people, telling others about the events experienced). Often, circumstantial and testimonial data do not exist; when they are available, they must however be supported by other objective elements that support and verify their reliability. One thinks, for example, of cases of suicide by multiple gunshots, cases of homicide in gunfights, or even cases where a single gunshot caused death not immediately but during the transport of the wounded person to hospital, so that a delay in rescue can be assumed. Let us also recall events such as double suicide or murder–suicide in which it appears difficult but indispensable to recognise the exact dynamics and succession of the acts performed because of the implications that may arise in both the criminal and civil spheres (compensation for possible damages) in the event that one of the two subjects survives (commorient–premorient).

Since haemorrhage is responsible for up to 40 percent of trauma-related deaths, it is important to define the underlying pathogenetic mechanism, but it is also essential to establish, with a good degree of approximation, the time span in which death occurred, starting from the moment of the fatal injury. For a correct dynamic reconstruction of the accident, it is useful, for example, to understand whether the subject died immediately after the criminal action or at a later time and was therefore able to be rescued. It is equally useful to be able to understand whether the death is causally linked to the aforementioned criminal action or to a possible delay in rescue operations [6,7,8]. In this respect, there are very few studies in the literature; there are no real major case studies analysing survival times after a haemorrhage, but rather individual reports often linked to the action of firearms, which are in any case not useful for defining ranges that can be used as a reference source for forensic practice.

In forensic science, one of the most discussed elements is the time of death [7]. The aim of this study is to provide, using the mathematical theorem, a valid model to provide an accurate time interval of death in traumatic cases with thoracic and/or abdominal trauma involving vascular injuries.

## 2. Materials and Methods

Through angiographic studies, it was possible to catalogue the calibres of the major arterial vessels. In particular, we used an extensive literature review of distributed one-dimensional models of the systemic arterial tree as a reference in order to be as accurate as possible [9]. For a given artery *a*, its particular flow rate $Q_{a}$ determines the volume ΔV flowing through the vessel in a time Δt as

(1) $$\Delta V=Q_{a}\Delta t.$$

The so-called Estimated Blood Volume (EBV) is a value that estimates the amount of blood in an individual by correlating it to their body weight. It is generally based on three factors: the person’s weight, gender and age. In this study, EBV is calculated using software prepared by the American Society of Anesthesia, given a person’s weight and height. Therefore, since it can be estimated that death from hypovolaemic shock occurs after a person loses 40% of their EBV, the time between receiving an injury at a single artery *a* and the death of the affected individual can be evaluated as

(2) $$T=0.4\frac{EBV}{Q_{a}}.$$

The flow rate of an artery can be assessed by considering the Hagen–Poiseuille (HP) law

(3) $$\Delta p_{a}=R_{a}Q_{a},$$

where $\Delta p_{a}$ is the pressure difference between the two ends of the vessel and $R_{a}$ is the resistance, expressed in $\mathrm{Pa}\cdot s\cdot m^{-3}$, determined by

(4) $$R_{a}=\frac{8\eta \ell_{a}}{\pi r_{a}^{4}},$$

where $\eta =2.084\times 10^{-3}\;\mathrm{Pa}\cdot s$ is the viscosity of the blood, $\ell_{a}$ is the length of the artery and $r_{a}$ is its section radius (both expressed in meters).

The cardiovascular systemic circulation can be represented as a closed circuit made up of different districts (aorta, arteries, arterioles, capillaries, venules, veins, vena cava) connected in series, i.e., crossed by the same total blood flow. Each district is formed by similar vessels connected in parallel, that is, with approximately the same difference in blood pressure at their ends. Since we are interested in arterial wounds, let us focus on the series formed by the first two districts crossed by the same blood flow, which is the total flow injected by the heart into the aorta with frequency $Q_{\mathrm{tot}}$. By applying the HP law to the aorta and the arterial district, we obtain

(5) $$\begin{array}{cc} \Delta p_{\mathrm{aorta}} & =R_{\mathrm{aorta}}Q_{\mathrm{tot}}, \end{array}$$

(6) $$\begin{array}{cc} \Delta p_{\mathrm{arteries}} & =R_{\mathrm{arteries}}Q_{\mathrm{tot}}, \end{array}$$

where $\Delta p_{\mathrm{arteries}}$ is the pressure difference between the extremities of the arterial district, characterised as a whole by the equivalent resistance $R_{\mathrm{arteries}}$. The resistance $R_{\mathrm{aorta}}$ of the aorta and the resistance $R_{\mathrm{arteries}}$ of the arteries represent 4% and 21% of the total systemic resistance, respectively [10]. So,

(7) $$R_{\mathrm{arteries}}=5.25\;R_{\mathrm{aorta}}$$

and

(8) $$\Delta p_{\mathrm{arteries}}=5.25\;R_{\mathrm{aorta}}Q_{\mathrm{tot}}.$$

Since the pressure difference is approximately the same, coinciding with $\Delta p_{\mathrm{arteries}}$, at the ends of each branch of the arteries that arise from the aorta, we can estimate the flow through a wound artery as

(9) $$Q_{a}=\frac{\Delta p_{\mathrm{arteries}}}{R_{a,\mathrm{tot}}}=5.25\frac{R_{\mathrm{aorta}}}{R_{a,\mathrm{tot}}}Q_{\mathrm{tot}},$$

where $R_{a,\mathrm{tot}}$ is the equivalent resistance of the series formed by the arterial segments connecting the aorta with the injured artery *a*. In the case of the aorta, instead, $Q_{a}=Q_{\mathrm{tot}}$ is considered. The calculated flow rate can then be inserted into the expression of *T* to determine the time required to reach a hypovolaemic shock condition.

The proposed approach can be generalised to the case of multiple injured arteries $\left(a_{1},a_{2},\ldots ,a_{n}\right)$, which are connected with the aorta without any segment in common. Since the volume ΔV flowing through these vessels in a time Δt in ordinary conditions is equal to

(10) $$\Delta V=\left(Q_{a_{1},\mathrm{tot}}+Q_{a_{2},\mathrm{tot}}+\cdots +Q_{a_{n},\mathrm{tot}}\right)\Delta t,$$

if total blood loss in a time Δt is neglected, the time to reach hypovolaemic shock can be evaluated as

(11) $$T=0.4\frac{EBV}{Q_{a_{1},\mathrm{tot}}+Q_{a_{2},\mathrm{tot}}+\cdots +Q_{a_{n},\mathrm{tot}}}.$$

Since we are interested in estimating the time of death, this calculation is sufficient for the purpose, as more accurate calculations would result in a greater risk of error.

## 3. Results

Penetrating wounds increase the risk of injury to major vessels and therefore of massive haemorrhage. The concept of the distribution of mortality after injury along a chronological axis was first characterised by Mullins et al., who described the trimodal distribution of death by injury by classifying deaths occurring in the immediate, early and late time periods after injury [11]. The basis of our experimental work is to apply the formulas of the flow rate of the affected vessel and the time relative to the amount of blood (leaking from the section of a given vessel and necessary to determine the state of hypovolaemic shock) in an attempt to calculate, obviously with approximation, the survival time before death. We have therefore analysed four different cases that have come to our attention, in which the injury of a single arterial vessel and the resulting haemorrhage caused the death of the victim, in order to test the validity of the physical method prepared, applying the theoretical principles described above. In the four cases, the judge requested an autopsy examination and determination of the time of death. Our study then used the results obtained from the autopsy investigations at the express request and authorisation of the judge.

In particular, we took as our starting point (with flow rate $Q_{a}=Q_{0}$) the main vessel of our organism, called the aorta, in its ascending tract. Other general data we have used are:

- Resistance of the aorta: $R_{\mathrm{aorta}}=0.19\cdot 10^{6}\;\mathrm{Pa}\cdot s\cdot m^{-3}$;
- Blood viscosity: $\eta =2.084\times 10^{-3}\;\mathrm{Pa}\cdot s$;
- Total flow rate evaluated as EBV/(60s), with EBV expressed in $m^{3}$.

The resistance of the cut artery is strongly dependent on the distance between the cut and the proximal end of the vessel, which is the only part where blood can flow after the injury. Since we miss this type of data in the considered case studies, we will assume that the cut occurs in the middle of the artery. Therefore, the cut artery provides a contribution $R_{a}/2$ to the total series resistance $R_{a,\mathrm{tot}}$. This assumption is necessitated by the lack of data: it would be very useful to know precisely the distance between the proximal end of the severed artery and the wound, because the flow depends crucially on the part of the artery travelled by the blood before it comes out. In particular, in Case 1, the estimated time for hypovolaemic shock is about 4 min if the wound is close to the proximal end, and more than 11 h if it is close to the distal end of the artery in question. The artery parameters are taken from Ref. [9]. The radius of the artery is estimated by averaging the proximal and distal lumen diameters. Based on these preliminary steps, we were able to perform the calculations for each individual case.

### 3.1. Case 1

**Figure 1:**
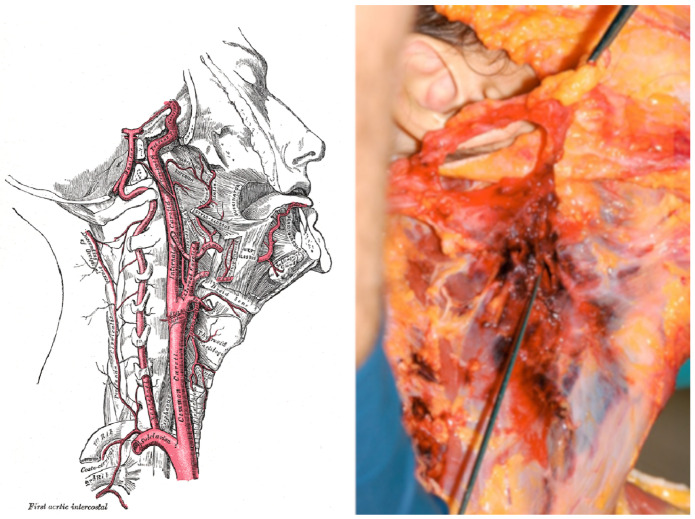
Anatomic representation of external carotid artery (**left panel**, figure from https://it.wikipedia.org/wiki/Arteria_carotide_esterna, accessed on 10 January 2023); external carotid artery injury (**right panel**) in the subject.

The first case studied concerns a woman aged 21, weighing 51 kg and measuring 164 cm in height, found dead in her flat, after having been sexually assaulted, who presented a main lesion (considering the wound as ’main’ as it was presented by the other superficial features, which were therefore not considerable to the study at hand) in the left laterocervical region 8 cm below and 1.5 cm in front of the external acoustic meatus (see Figure 1). The lesion measured 8 cm, was arranged obliquely in a caudal and lateral direction, and was extensively damaged, with exposure of the underlying tissues that appeared dissected down to the osteocartilaginous plane. The means used to produce this lesion was identified as a blade belonging to a point and cutting weapon capable of penetrating tissue up to a depth of 8 cm. The injury just described constituted a large 8×4 cm breach in the left sternocleidomastoid muscle that deepened to the right. The weapon used had severed the external carotid artery through its tissue. Knowing the victim’s weight and height, we were able to calculate, using the EBV formula, the woman’s total blood volume (this calculation was performed using specific software available on the American Society of Anaesthesia websites).

Cut vessel: Left superior thyroid artery (external carotid 1–13).

- Considered branch: 3, 5, 13;
- $\ell_{3}=34$ mm, $\ell_{5}=94$ mm, $\ell_{1}3=41$ mm;
- $r_{3}=\left(20.2+18.0\right)/4$ mm, $r_{5}=\left(13.5+7.0\right)/4$ mm, $r_{1}3=\left(5.0+4.5\right)/4$ mm;
- $R_{3}=2.17\times 10^{4}\;\mathrm{Pa}\cdot s\cdot m^{-3}$, $R_{5}=7.24\times 10^{5}\;\mathrm{Pa}\cdot s\cdot m^{-3}$, $R_{13}=6.84\times 10^{6}\;\mathrm{Pa}\cdot s\cdot m^{-3}$.

Therefore, considering the ATLS table we calculated

(12) $$EBV=3640\;\mathrm{mL}=3.64\times 10^{-3}\;m^{3}$$

Thus, we obtain

(13) $$\begin{array}{cc} Q_{\mathrm{tot}} & =\frac{EBV}{1\;\min}=6.1\times 10^{-5}\;m^{3}\cdot s^{-1}, \end{array}$$

(14) $$\begin{array}{cc} Q_{a} & =5.25\;\frac{R_{\mathrm{aorta}}}{R_{3}+R_{5}+R_{13}/2}Q_{\mathrm{tot}}=1.46\times 10^{-7}\;m^{3}\cdot s^{-1}. \end{array}$$

By applying the formula to calculate the time, we obtain

(15) $$T=0.4\frac{EBV}{Q_{a}}=167\;s=2.8\;\min.$$

The obtained result of 167 s thus corresponds to the time in which 40 percent of the blood leaked from the left upper thyroid artery, causing a state of shock and thus the death of the woman. If it is assumed that, during the attack, the subject moved or attempted to escape, the muscular effort must be taken into account. During this escape phase, the subject increases the cardiac output by up to 4 times [12]. Consequently, if we want to apply this to the calculations we have just made, we will have that

(16) $$t=\frac{T}{4}=42\;s.$$

By comparing the result obtained with the approximate calculations of the time of death provided by the expert following the autopsy examination, on the basis of other parameters, it is possible to evaluate the reliability of our experimental study.

Since the dynamics of the event, i.e., whether and how many movements the victim could have made after the trauma, are not known unequivocally, we consider a time interval between 70 s (subject in full movement) and 2.8 min (subject immobilised) to be more appropriate. The result of the autopsy, performed on the corpse of the young woman, established that she was first a victim of asphyxiation (suffocation), then fatally struck by the point-and-shoot weapon that severed the thyroid artery in the upper left side. The exit occurred due to the combination of blood loss (haemorrhage) and the simultaneous blockage of the airway due to blood penetration (in a comatose state the cough reflex is notoriously absent), resulting in asphyxiation. In the present case, based on the relevance of the classical parameters used in the medico-legal field, an agony time of 1–10 min was estimated (total time in which the assault, fatal injury and death of the victim occurred). The value previously obtained on the basis of our experimental calculations and relating exclusively to the woman’s survival time following the cutting of the left upper thyroid artery can safely be considered consistent with the reality of the facts and within the range proposed by the medico-legal consultant.

### 3.2. Case 2

**Figure 2:**
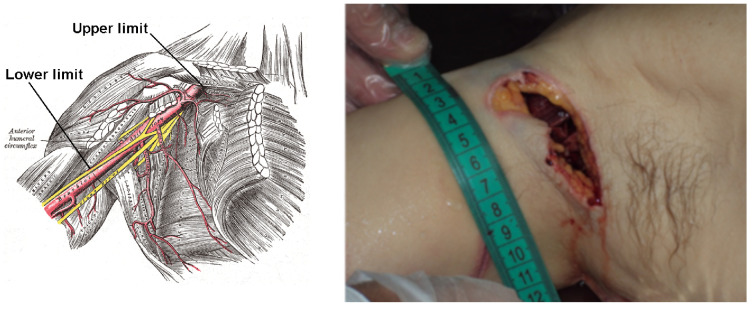
Anatomic representation of right axillary artery (**left panel**, figure from https://en.wikipedia.org/wiki/Axillary_artery, accessed on 10 January 2023); right axillary artery injury (**right panel**) in the subject.

The second case examined concerned a 70-year-old man, weighing 65 kg and measuring 168 cm in height. The subject was at home with their granddaughter, intent on repairing the boiler on the balcony of their house, when he suddenly lost balance, breaking the glass of the bathroom window and causing an extensive laceration of the armpit vessels. He then moved inside the house in search of help, passing through the kitchen, the corridor and finally falling to the floor in the entrance of the house. Therefore, the emergency staff, promptly alerted by their niece, who had arrived on the scene, pronounced their death at 5:20 p.m. due to “haemorrhagic shock from excision of the right axillary artery”. On subsequent external examination, we noted at the right axillary cavity the presence of a deep stitch and a stab wound extending in the sagittal plane for a length of 7 cm, deepening similar to a clarinet beak, with an upward and lateral–medial inclination (see Figure 2). In the lower part of the lesion, a large excision of the axillary artery and vein could be appreciated. Since the man’s weight and height are known, we were able to calculate the subject’s total blood volume using the EBV formula.

Cut vessel: Right axillary artery (subclavian B, axillary, brachial-7).

- Considered branch: 3, 4, 7;
- $\ell_{3}=34$ mm, $\ell_{4}=34$ mm, $\ell_{7}=422$ mm;
- $r_{3}=\left(20.2+18.0\right)/4$ mm, $r_{4}=\left(11.5+9.0\right)/4$ mm, $r_{7}=\left(8.1+4.7\right)/4$ mm;
- $R_{3}=2.17\times 10^{4}\;\mathrm{Pa}\cdot s\cdot m^{-3}$, $R_{4}=2.62\times 10^{5}\;\mathrm{Pa}\cdot s\cdot m^{-3}$, $R_{7}=2.14\times 10^{7}\;\mathrm{Pa}\cdot s\cdot m^{-3}$.

Therefore, considering the ATLS table we calculated

(17) $$EBV=4840\;\mathrm{mL}=4.84\times 10^{-3}\;m^{3}$$

Thus, we obtain

(18) $$\begin{array}{cc} Q_{\mathrm{tot}} & =\frac{EBV}{1\;\min}=8.1\times 10^{-5}\;m^{3}\cdot s^{-1}, \end{array}$$

(19) $$\begin{array}{cc} Q_{a} & =5.25\;\frac{R_{\mathrm{aorta}}}{R_{3}+R_{4}+R_{7}/2}Q_{\mathrm{tot}}=7.34\times 10^{-6}\;m^{3}\cdot s^{-1}. \end{array}$$

By applying the formula to calculate the time, we obtain

(20) $$T=0.4\frac{EBV}{Q_{a}}=264\;s=4.4\;\min.$$

The result obtained in minutes therefore corresponds to the time in which 1.9 litres of blood (corresponding to 40% of the total volume of blood, calculated with the EBV formula, the loss of which leads to a state of hypovolaemic shock) escaped from the right artery axillary, causing a state of shock and therefore the death of the subject. If one assumes that, during the accident, the subject moved or attempted to escape, muscular effort must be considered. During this escape phase, the subject increases cardiac output up to 4 times [12]. Consequently, if we apply this to the calculations we just performed, we obtain

(21) $$t=\frac{T}{4}=1.1\;\min.$$

Furthermore, in this case, not really knowing the precise dynamics of the events nor how many movements the victim was able to perform after the injury, we believe that a time interval between 1.1 (subject in full motion), even if empirical, and 4.4 min (immobilised subject) is more appropriate. Even this result, although it cannot be considered representative of the exact reality, can be considered useful as an indicative reference of the victim’s survival time. Comparing, therefore, the result obtained with the approximate calculations of the time of death provided by the expert following the autopsy examination, on the basis of other parameters, in this case, it was also possible to evaluate the reliability of our experimental study.

### 3.3. Case 3

**Figure 3:**
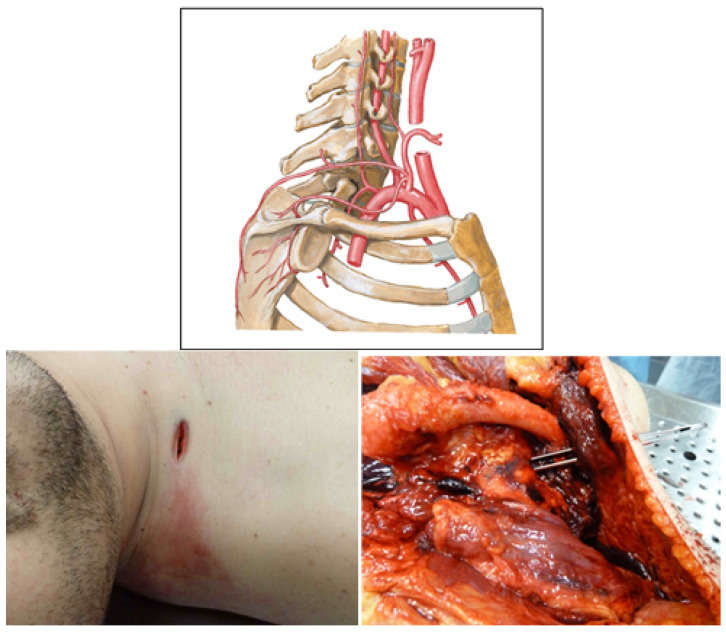
Anatomic representation of right subclavian artery (**top panel**, figure from https://www.medicinembbs.com/2011/04/arteries.html, accessed on 10 January 2023); skin injury (**bottom left panel**) and right subclavian artery injury (**bottom right panel**) in the subject.

We analysed the case of a 28-year-old male, weighing 97.6 kg and measuring 178 cm in height. The subject had been involved in a fight with several individuals, one of whom had fatally wounded him with a knife. A preliminary inspection from the cadaver revealed, in addition to the widespread blood stains present on the entire body surface of the victim, the presence of a tip and a cut wound of the size of 2.5×0.6, localised in the region of the right supraclavicular (see Figure 3). This lesion extended deep into the tissue planes until the right subclavian artery was severed.

Cut vessel: Right subclavian artery (subclavian A-4).

- Considered branch: 3, 4;
- $\ell_{3}=34$ mm, $\ell_{4}=34$ mm;
- $r_{3}=\left(20.2+18.0\right)/4$ mm, $r_{4}=\left(11.5+9.0\right)/4$ mm;
- $R_{3}=2.17\times 10^{4}\;\mathrm{Pa}\cdot s\cdot m^{-3}$, $R_{4}=2.62\times 10^{5}\;\mathrm{Pa}\cdot s\cdot m^{-3}$.

Therefore, considering the ATLS table, we calculated

(22) $$EBV=7275\;\mathrm{mL}=7.28\times 10^{-3}\;m^{3}$$

Thus, we obtain

(23) $$\begin{array}{cc} Q_{\mathrm{tot}} & =\frac{EBV}{1\;\min}=1.2\times 10^{-4}\;m^{3}\cdot s^{-1}, \end{array}$$

(24) $$\begin{array}{cc} Q_{a} & =5.25\;\frac{R_{\mathrm{aorta}}}{R_{3}+R_{4}/2}Q_{\mathrm{tot}}=7.9\times 10^{-4}\;m^{3}\cdot s^{-1}>Q_{\mathrm{tot}}⟹Q_{a}=Q_{\mathrm{tot}}. \end{array}$$

By applying the formula to calculate the time, we obtain

(25) $$T=0.4\frac{EBV}{Q_{a}}=24\;s=0.4\;\min.$$

The equation obtained with the considered approximations overestimates the value of $Q_{a}$, which is even greater than the total flow rater. In this case, we consider $Q_{\mathrm{tot}}$ as an estimate of $Q_{a}$, as if the aorta were injured. This is reasonable, as a very small and low-resistance tract connects the wound to the first segment of the aorta.

Furthermore, in this case, the final considerations proposed for the previous cases are valid. If it is to be assumed that, during the attack, the subject moved or attempted to escape, muscular effort must be considered. During this escape phase, the subject increases cardiac output up to 4 times [12]. Consequently, if we want to apply this to the calculations we just made, we obtain

(26) $$t=\frac{T}{4}=6\;s.$$

By comparing the result obtained with the approximate calculations of the time of death provided by the expert following the autopsy examination, on the basis of other parameters, it was possible to evaluate the reliability of our experimental study. Since also in this case the precise dynamics of the event are not certain, i.e., it is not known how many movements the victim could make after the injury, the estimated range is between 6 (subject in full motion) and 24 s (immobilised matter). However, the data obtained, even applying the aforementioned theory, are compatible with the tanatochronic data obtained during the cadaver examination and during the judicial inspection.

### 3.4. Case 4

**Figure 4:**
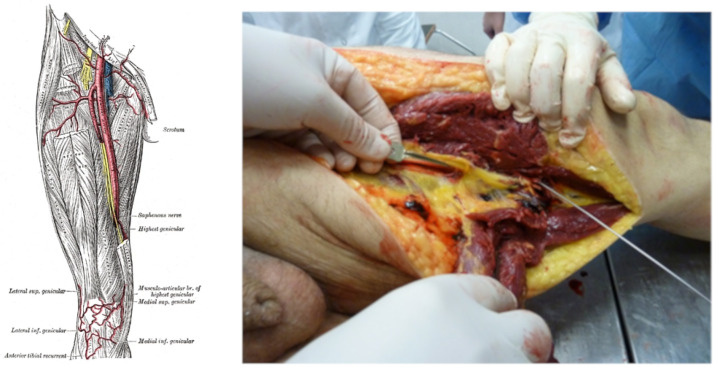
Anatomic representation of femoral artery (**left panel**, figure from https://radiopaedia.org/articles/femoral-artery, accessed on 10 January 2023); femoral artery injury (**right panel**) in the subject.

Finally, we considered the case of a man aged 50, weighing 98 kg and with a height of 173 cm. The subject was killed from a gunshot wound to the left lower limb (thigh). This lesion extended deep into the tissue planes until it severed the left femoral artery (see Figure 4). The damaging event took place at 4:45 p.m. on a February day and the death was registered at 5:05 p.m. on the same day.

Cut vessel: Left femoral (femoral–46).

- Considered branch: 42, 44, 46;
- $\ell_{42}=59$ mm, $\ell_{44}=144$ mm, $\ell_{46}=443$ mm;
- $r_{42}=\left(7.9+7.0\right)/4$ mm, $r_{44}=\left(6.4+6.1\right)/4$ mm, $r_{46}=\left(5.2+3.8\right)/4$ mm;
- $R_{42}=1.63\times 10^{6}\;\mathrm{Pa}\cdot s\cdot m^{-3}$, $R_{44}=8.01\cdot 10^{6}\;\mathrm{Pa}\cdot s\cdot m^{-3}$, $R_{46}=9.18\times 10^{7}\;\mathrm{Pa}\cdot s\cdot m^{-3}$.

Therefore, considering the ATLS table, we calculated

(27) $$EBV=7350\;\mathrm{mL}=7.35\times 10^{-3}\;m^{3}$$

Thus, we obtain

(28) $$\begin{array}{cc} Q_{\mathrm{tot}} & =\frac{EBV}{1\;\min}=1.2\times 10^{-4}\;m^{3}\cdot s^{-1}, \end{array}$$

(29) $$\begin{array}{cc} Q_{a} & =5.25\;\frac{R_{\mathrm{aorta}}}{R_{42}+R_{44}+R_{46}/2}Q_{\mathrm{tot}}=2.20\times 10^{-6}\;m^{3}\cdot s^{-1}. \end{array}$$

By applying the formula to calculate the time, we obtain

(30) $$T=0.4\frac{EBV}{Q_{a}}=1.34\times 10^{5}\;s=22.2\;\min.$$

Furthermore, in this case, the final considerations proposed for the previous cases are valid. If it is to be assumed that, during the attack, the subject moved or attempted to escape, muscular effort must be considered. During this escape phase, the subject increases cardiac output up to 4 times [12]. Consequently, if we want to apply this to the calculations we have just made, we would have

(31) $$t=\frac{T}{4}=5.55\;\min$$

Therefore, by comparing the result obtained with the data present in the documents (injury occurred at 4:45 p.m. and death observed at 5:05 p.m.), it was possible to evaluate the reliability of our experimental study.

## 4. Discussion

All forensic scientists know that every dead person ‘talks’; however, unfortunately, sometimes the appropriate tools to understand this do not exist. Estimating the time of death with specific indications of the victim’s survival time is a frequent request made to forensic scientists by judges [13]. In cases of death by profuse haemorrhage, defining the time interval between the injury of an (arterial) vessel and death can help to obtain more precise information about the dynamics of the event. For example, understanding whether the victim survived for some time and performed autonomous acts can serve to assess the reliability of a witness and the reconstruction of the events provided.

The aim of this scientific work is to create a tool for calculating the time (survival from injury to death) based on mathematical formulae and the principles of applied physics, complementing the methods used to estimate the time of death. Livor mortis, rigor mortis and cadaveric temperature are indeed the classic signs of death that, combined with intrinsic and extrinsic factors, are generally used to determine the time of death [14]. However, these signs do not allow us to understand whether and how long the victim survived after the injury. Through the study of hypostasis, cadaveric rigidity and body temperature, we can, in fact, establish the time of death over a wide time range. If there are no other certain circumstantial data (e.g., video recordings), it will not be possible to provide a detailed reconstruction. Knowledge of the survival interval calculated from the time of haemorrhage of the arteries could, in fact, be a further parameter to be assessed in the context of forensic investigations.

While we are aware that forensic medicine, similar to all branches of medicine, suffers from the extreme variability of science and human biology in its assessments, trying to find methods that can reduce uncertainty may be useful in reconstructing events [15]. Shock is a phenomenon of a biological nature that is expressed according to a variable reaction of the organism to traumatic stress. This response to the traumatic event cannot therefore be encapsulated in formulas or schemes, as individual factors that also depend on the subject’s age, physical state and somatic constitution contribute to its onset and extent [16]. These factors, which cannot be quantified, must be considered ‘imponderables’ and are responsible for abnormal behaviour that can sometimes be observed in subjects who are capable of performing complex acts, even after particularly severe traumatic punctures or major haemorrhages [17,18,19,20,21]. The same factors, however, not only affect the ‘variability’ of the evolution of shock but also bring about changes, which are sometimes substantial, in the study of cadaveric phenomenology. All forensic and necropsy medicine disciplines cannot and should not be considered a certain science based on known elements; instead, they are modifiable through imponderable influences [22,23]. The method applied is an attempt to parameterise an assessment that is undoubtedly difficult and influenced, as already mentioned, by factors that are often not exactly assessable; however, the results obtained, relating to only four cases, seem to confirm what emerged on the basis of the other elements currently used in the medico-legal sphere to reconstruct events, in addition to coinciding with what is present in the medical documentation and witness data [24].

## 5. Conclusions

It is essential to be aware of the intrinsic impact of the experimental methods proposed to improve the accuracy of information on the victim’s survival time. Indeed, the deductions obtained in this way are more free from estimations related to the subjectivism of the examiner; therefore, we believe that they are better used in the context of an overall case evaluation. The work we have proposed is a pilot study for future work. In fact, we intend to improve the data by extending the case and statistical analysis with particular attention paid to interference factors in order to confirm their actual usability in forensic work. We intend to extend this study by also investigating cases in which more than one artery is injured. Subsequently, this calculation may be expanded to include other traumatic injuries that produce haemorrhage.

## Data Availability

Data are contained within the article.

## Abbreviations

The following abbreviations are used in this manuscript:

EBV	Estimated Blood Volume
